# Spontaneous Heparin-Induced Thrombocytopenia and Venous Thromboembolism following Total Knee Arthroplasty

**DOI:** 10.1155/2017/4918623

**Published:** 2017-02-05

**Authors:** Kevin Baker, Ming Y. Lim

**Affiliations:** ^1^Department of Medicine, Medical University of South Carolina, Charleston, SC, USA; ^2^Department of Medicine, Division of Hematology/Oncology, Medical University of South Carolina, Charleston, SC, USA

## Abstract

A 72-year-old Caucasian woman was admitted for an elective left total knee arthroplasty. Her surgery was uncomplicated and she was discharged to a rehabilitation facility. Twelve days later, she developed acute shortness of breath followed by a syncopal episode. She was hypoxic and cyanotic, requiring hospitalization and intubation, and was subsequently diagnosed with bilateral submassive pulmonary emboli and bilateral lower extremity deep vein thrombosis. She was started on unfractionated heparin infusion. Within 24 hours of exposure, she had an acute decrease in platelet count to 48,000. Heparin was discontinued and argatroban was initiated due to concern for heparin-induced thrombocytopenia (HIT). Both quantitative enzyme immunoassay and functional assay confirmed the diagnosis of HIT. The patient had no prior lifetime heparin exposure. Given the absence of preceding heparin therapy, this case is consistent with the diagnosis of spontaneous HIT.

## 1. Introduction

The evaluation of thrombocytopenia and thrombosis can be broad and the patients' work-up is typically determined based on their clinical presentation, medication exposure, and past medical history. However, patients often do not display all the prerequisite signs and symptoms of a disease process. This should lead a clinician to hold a high index of suspicion for syndromes with high morbidity and mortality.

Heparin-induced thrombocytopenia (HIT) is a prothrombotic disorder that carries significant morbidity and mortality if not properly identified and treated with anticoagulation. There have been rare cases of spontaneous HIT which is the clinical syndrome of HIT without prior heparin exposure. The lack of heparin exposure can lead to significant delays in the diagnosis of spontaneous HIT. The case below illustrates the work-up, diagnosis, and management of spontaneous HIT and the importance of maintaining a broad differential when investigating thrombocytopenia and thrombosis.

## 2. Case Presentation

A 72-year-old Caucasian woman with a past medical history significant for diabetes mellitus, hyperlipidemia, hypothyroidism, and osteoarthritis was admitted for an elective left total knee arthroplasty. Her medications included aspirin 81 mg daily, simvastatin 40 mg daily, glipizide 2.5 mg daily, iron sulfate 325 mg daily, vitamin D3 2000 units daily, and ezetimibe 10 mg daily. She was allergic to formaldehyde. Perioperatively, she received spinal anesthesia with bupivacaine and intravenous propofol, phenylephrine, midazolam, epinephrine, cefazolin, and vancomycin. Notably, no heparin was given, no blood reinfusion or salvage devices were used, and no central venous or arterial catheters were used intraoperatively.

Postoperatively, her hospital medications included ondansetron, metoclopramide, diphenhydramine, acetaminophen, and morphine. On postoperative day (POD) 1, laboratory results revealed white blood cell count (WBC) of 12.9 K/mL (range: 3.7–10.1 K/mL), hemoglobin (Hgb) 10.8 g/dL (range: 11.5–15.4 g/dL), platelets 179 K/mL (range: 156–352 K/mL) sodium 143 mmol/L (range: 136–145 mmol/L), potassium 4.0 mmol/L (range: 3.5–5.1 mmol/L), chloride 107 mmol/L (range: 97–107 mmol/L), bicarbonate 26 mmol/L (range: 21–32 mmol/L), blood urea nitrogen (BUN) 8 mg/dL (range: 7–18 mg/dL), and creatinine 0.7 mg/dL (range: 0.6–1.3 mg/dL). She was continued on her home medications with the addition of gabapentin, celecoxib, oxycodone, and pantoprazole. Her home aspirin was stopped and she was started on aspirin 325 mg twice daily for deep vein thrombosis (DVT) prophylaxis. She was discharged on POD3 to a rehabilitation center for physical therapy. Her laboratory results on discharge were WBC 11 K/mL, Hgb 9.9 g/dL, and platelets 183 K/mL.

On POD12, while ambulating with a walker at the rehabilitation center, she developed acute shortness of breath and subsequently lost consciousness. The patient was found to be hypotensive at 105/37 mmHg, tachycardiac at 117, having respiratory rate of 33, and oxygen saturation of 80% on pulse oximeter. She was transported to the nearest hospital.

On arrival to the hospital, she was unresponsive and cyanotic with a blood pressure of 80/57 mmHg, pulse of 123, respiratory rate of 38, and oxygen saturation of 86%. She was intubated and placed on vasopressors. Initial work-up was significant for WBC 22.1 K/mL, Hgb 10.4 g/dL, platelets 85 K/mL, sodium 136 mmol/L, potassium 4.0 mmol/L, chloride 102 mmol/L, bicarbonate 18 mmol/L, BUN 12 mg/dL, creatinine 1.1 mg/dL, and calcium 8.6 mg/dL. Computed tomography (CT) angiography of the chest revealed bilateral pulmonary emboli. A transthoracic echocardiogram (TTE) showed an ejection fraction of 72% with a dilated hypocontractile right ventricle, mild right atrial enlargement, and right ventricular systolic pressure (RVSP) of 45–50 mmHg. She had no prior history of heart failure or pulmonary hypertension. She was initiated on unfractionated heparin infusion for treatment of pulmonary embolism. Repeat laboratory testing 14 hours later showed a subsequent decrease in platelet count to 48 K/mL. She was then transferred to our institution (a tertiary care facility) for further management of her hypoxic respiratory failure secondary to pulmonary embolism, cardiogenic shock, and thrombocytopenia.

On arrival at our institution, laboratory testing revealed WBC 19.6 K/mL, Hgb 7.2 g/dL, platelets 25 K/mL, d-dimer >20 *μ*g/mL (range: <0.43 *μ*g/mL), fibrinogen 315 mg/dL (range: 231–486 mg/dL), haptoglobin 154 mg/dL (range: 14–258 mg/dL), lactate dehydrogenase (LDH) 427 u/L (range: 100–240 u/L), prothrombin time (PT) 17.2 s (range: 12–15.1 s), and activated partial thromboplastin time (aPTT) 135.7 s (range: 23.3–35.7 s). Due to concern for heparin-induced thrombocytopenia (HIT), unfractionated heparin was discontinued. She received 1 unit of packed red blood cells (pRBC) for anemia and was started on intravenous argatroban with a goal aPTT of 50–80 s. Pulmonary angiogram demonstrated extensive clot in the right middle and lower arteries and left lower artery. Given hemodynamic instability, she underwent catheter-directed thrombolysis with alteplase at 1 mg/hour. Bilateral upper and lower extremity duplex ultrasounds revealed thrombosis in the superficial left cephalic, left common femoral, left popliteal, and right posterior tibial vein.

A hypercoagulable work-up, which included lupus anticoagulant, dilute russell viper venom time, anticardiolipin IgG and IgM, and beta-2-glycoprotein IgG and IgM antibodies, was negative. Testing for platelet factor 4 (H/PF4) antibodies using IgG-specific enzyme immunoassay (EIA) (Immucor) was strongly positive at 2.971 optical density (OD) units (normal range: <0.399 OD units). Subsequent serotonin release assay testing (BloodCenter of Wisconsin, Inc.) showed 100% release with low-dose heparin (0.1 U/mL) and 1% release at high dose heparin (100 U/mL), which was strongly consistent with the diagnosis of HIT.

The patient was continued on intravenous argatroban infusion and alteplase for approximately 36 hours. Alteplase was stopped on POD 14 due to platelet count and fibrinogen nadir of 14 K/mL and 152 mg/dL, respectively. The patient was also hemodynamically stable without vasopressors at this point. Repeat TTE postcatheter-directed thrombolysis demonstrated a modest improvement in RVSP and right ventricular systolic function and dilation. Unfortunately, on POD 16 the patient developed worsening neurologic exam with decreased alertness and right upper extremity weakness. CT of the head without contrast demonstrated left thalamic and parietal hematoma, scattered subarachnoid hemorrhage in the posterior left cerebral hemisphere, vasogenic edema, and mass effect with a 2 mm left-to-right midline shift without herniation. Due to the patient's intracerebral hemorrhage, argatroban was stopped, and a retrievable Denali inferior vena cava filter was placed via the right femoral vein. Argatroban had been within therapeutic range except for one supratherapeutic level (101 s) 24 hours prior to the onset of neurologic symptoms and alteplase had been stopped for 36 hours. Neurosurgical intervention was not felt to be beneficial in this situation. Instead, the platelet transfusion threshold was increased to 50 K/mL. She remained off anticoagulation therapy over the following 7 days. Her Hgb stabilized to 9.0 g/dL and her platelet count continued to improve to 148 K/mL on POD 22.

The neurosurgery service felt comfortable restarting anticoagulation therapy at a prophylactic dose 1-week postintracranial hemorrhage, on POD 23. As there was no suitable alternative low-dose or prophylactic anticoagulants approved for HIT, a discussion with the patient and family was initiated regarding the risk of intracranial hemorrhage with resumption of therapeutic anticoagulation therapy versus the risk of recurrent thrombosis off anticoagulation therapy.

With the patient and family's consent, intravenous argatroban infusion was restarted with a lower aPTT goal of 45–60 s. Over the following two weeks, she remained neurologically stable, with no further evidence of bleeding on repeat radiological imaging. Her platelet count continued to improve to 198 K/mL on POD 28. At this time, she was bridged to warfarin in preparation for outpatient anticoagulation management. There were concerns about compliance with warfarin monitoring and significant difficulties titrating her warfarin dosing over the next 2 days due to the effect of argatroban on International Nationalized Ratio (INR) levels. Given this, she was switched to apixaban 5 mg twice daily on POD 30 with platelet count of 188 K/mL and discharged to complete 3 months of therapy with recommendations to follow up with her local primary care physician. Unfortunately, the patient was lost to follow-up and long-term outcomes regarding further thrombosis or hemorrhage were unable to be monitored.

## 3. Discussion

Heparin-induced thrombocytopenia (HIT) is a prothrombotic disorder where heparin, a polyanion molecule, acts as a hapten when associated with PF4 causing IgG-mediated platelet activation. Activation of platelets leads to thrombosis and thrombocytopenia. HIT can manifest with signs and symptoms such as fevers, chills, tachycardia, hypoxia, and venous or arterial thrombosis [1, 2]. HIT typically occurs within 5–10 days from the patient's first heparin exposure. If there has been prior heparin exposure within the last 30 days, patients can develop rapid onset HIT which is caused by interaction of heparin/PF4 complexes with antibodies formed during their last heparin exposure. In this situation, the interaction of heparin with preexisting antibodies and the development of signs or symptoms occur within hours of heparin reexposure.

Initial evaluation utilizes a quantitative enzyme immunoassay that measures antibodies binding to PF4-heparin complexes and has high sensitivity but low specificity [3, 4]. Many patients that are exposed to heparin develop antibodies which recognize heparin/PF4 complexes however not all antibodies cause platelet activation and the ensuing complications from thrombocytopenia and thrombosis. To avoid overdiagnosis, confirmatory testing of the patient's serum with a functional assay that detects platelet activation induced by these antibodies, such as the serotonin release assay, is usually required. High platelet activity on serotonin release assay at low levels of heparin (0.1 U/mL) and inhibition at high levels (100 U/mL) confirm the diagnosis of HIT.

Our patient had no prior lifetime exposure to heparin but developed rapid decline in platelet counts 14 hours after exposure to unfractionated heparin. There have been a few case reports describing spontaneous HIT, in which the clinical and serologic features of HIT are seen in these patients despite the absence of preceding heparin therapy, similar to the presentation of our patient [5–8]. The exact pathophysiology remains unclear but is thought to be due to the interaction of negative polyanions such as nucleic acids and lipopolysaccharides with PF4 [9, 10]. The development of antibodies to these polyanion complexes can trigger activation of HIT within hours after initial exposure to heparin. This can explain the affinity for some patients to develop spontaneous HIT following orthopedic procedures where there is significant tissue injury or ischemia from the use of tourniquets [5, 7]. Other causes such as gram-negative bacteremia have also been associated with spontaneous HIT [9, 10].

Treatment is aimed at stopping all sources of heparin and starting the patient on nonheparin therapeutic anticoagulants such as argatroban, lepirudin, or danaparoid, given that these patients are in a prothrombotic state [11]. Once the platelet count has returned to normal levels, patients are transitioned to vitamin K antagonist such as warfarin [4]. However there have been a small number of studies indicating that it may be safe and efficacious to place patients on direct oral anticoagulants (DOACs), which is what was started for this patient given the difficulties with warfarin dosing [12, 13].

In summary, this case adds to the limited literature on spontaneous HIT to create awareness that HIT can occur without prior heparin exposure.
